# Supplementary material for: Predation and fragmentation portrayed in the statistical structure of prey time series
Source: BMC Ecol. 2009 May 6;9:10. doi: 10.1186/1472-6785-9-10 (PMC2689204; doi:10.1186/1472-6785-9-10)
Supplement: Additional file 2 — Voles and related classes ODDox Documentation. ODDox documentation of the agent-based model (ALMaSS) applied by Hendrichsen et al. The documentation is started by activating main.html. [file 1472-6785-9-10-S2.zip › Vole_ODDox/hierarchy.html]

ALMaSS ODDox: Hierarchical Index

- Main Page
- Related Pages
- Classes
- Files

- Alphabetical List
- Class List
- Class Hierarchy
- Class Members

# Class Hierarchy

This inheritance list is sorted roughly, but not completely, alphabetically:

- AlleleFreq- AnimalPosition
    - RoeDeerInfo
    - CompareState- CompareX- CompareY- Crop
            - SetAside- WinterWheat
            - CropRotation- Farm
                - AgroChemIndustryCerealFarm1- AgroChemIndustryCerealFarm2- AgroChemIndustryCerealFarm3- ConventionalCattle- ConventionalPig- ConventionalPlant- ConvMarginalJord- NoPesticideBaseFarm- NoPesticideNoPFarm- OrganicCattle- OrganicPig- OrganicPlant- PesticideTrialControl- PesticideTrialToxicControl- PesticideTrialTreatment- UserDefinedFarm1- UserDefinedFarm10- UserDefinedFarm11- UserDefinedFarm12- UserDefinedFarm13- UserDefinedFarm14- UserDefinedFarm15- UserDefinedFarm16- UserDefinedFarm2- UserDefinedFarm3- UserDefinedFarm4- UserDefinedFarm5- UserDefinedFarm6- UserDefinedFarm7- UserDefinedFarm8- UserDefinedFarm9
                - FarmEvent- GeneticMaterial- GeneticMaterial1616- IntArray100- Population\_Manager
                          - TPredator\_Population\_Manager- Vole\_Population\_Manager
                          - probe\_data- rectangle- Rotation- Starter- struct\_Predator- struct\_Vole\_Adult- TALMaSSObject
                                        - TAnimal
                                          - TPredator
                                            - Owl- Weasel
                                            - Vole\_Base
                                              - Vole\_Female- Vole\_Male

---

Generated on Thu Jan 22 14:13:45 2009 for ALMaSS ODDox by 
 1.5.6
